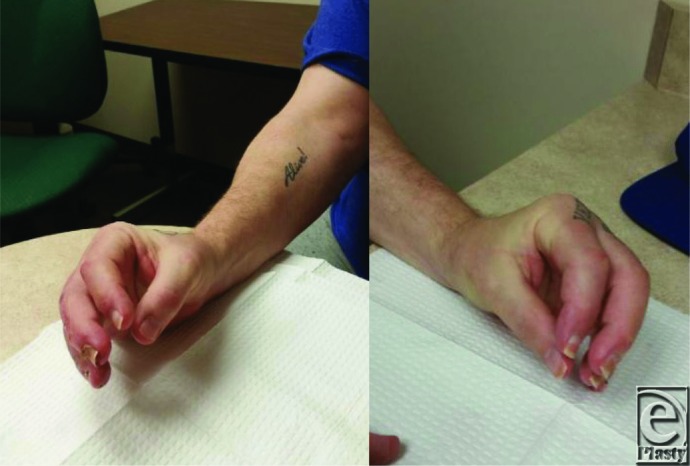# Bilateral Frostbite of the Hands

**Published:** 2015-07-07

**Authors:** T. Snoap, E. Gallagher, A. Snoap, T. Ruiter

**Affiliations:** ^a^Department of Orthopedics, Homer Stryker M.D. School of Medicine, Western Michigan University, Kalamazoo; ^b^Indiana University School of Medicine, Indianapolis; ^c^Borgess Medical Center, Kalamazoo, Mich

**Keywords:** frostbite, cold injury, amputation, digit, hypothermia

**Figure:**
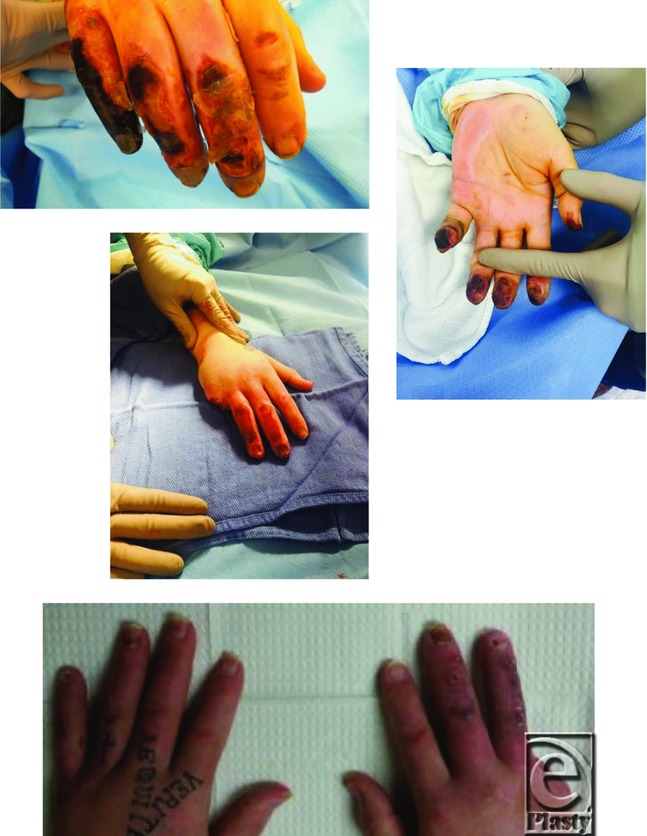


## DESCRIPTION

A 55-year-old man presented with frostbite injury to bilateral hands after losing consciousness outside during the winter season. There were signs of necrosis to all digits. After thorough debridement, all digits were salvaged, with the exception of his right small finger, which was amputated at the Metacarpophalangeal joint.

## QUESTIONS

1. **What people are at risk of developing frostbite?**
2. **How is frostbite injury classified?**
3. **What is the clinical presentation of frostbite?**
4. **What are the treatments of frostbite?**

## DISCUSSION

Frostbite injuries historically were seen most commonly in military personnel during times of war. In more recent years, these injuries have been seen more frequently in the general population. Frostbite is seen more in men than in women at a ratio of 10:1. The most commonly affected age group is 30 to 50 years.^1^ There have been several risk factors noted for frostbite including housing status, occupation, and recreational hobbies. Perhaps, the most important predisposing factor is the use of alcohol, which was in a study by Valnicek et al^2^ found to be associated with nearly 50% of inpatient hospitalizations for the diagnosis of frostbite.

The severity of damage to the tissues from frostbite injury can be classified into 4 degrees. First degree is reversible changes to the tissues. Second degree is associated with superficial dermal damage. Third degree involves deep dermal damage, and, finally, the fourth degree implicates damage to the subcutaneous tissue, muscles, nerves, and bone.^3^

Clinical manifestations of frostbite vary widely on the basis of time from injury as well as the depth of injury. In the acute stages, it is common for patients to have symptoms of numbness or lack of normal motor function. During the warming process, patients often experience an intense throbbing pain in the involved extremity. Physical examination can help differentiate superficial from deep injury. Deep injuries are more commonly associated with blisters, which can be clear or hemorrhagic. The very severe fourth-degree cold injuries can present with a blue to gray hue, with a hardened feeling to the skin with palpation. Eschar formation commonly occurs in the 9- to 15-day range, and mummification with clear demarcation occurs at 22 to 45 days.^4^

In general, a prognosis cannot be given before the tissues are allowed to thaw. In the early stages of injury, it is difficult to assess tissue viability and, often, surgical decision making must be delayed until clear demarcation of the injury zone is established. Emergent surgical treatment of frostbite is rarely necessary unless there is concern for infection or compartment syndrome. The first step in treatment is rewarming the damaged hand and correcting any associated hypothermia or hypovolemia. This process generally takes 15 to 30 minutes and is performed in water at 100.4°F to 104°F.^5^ Debridement of any ruptured blisters is recommended; however, the management of intact blisters has been debated in the literature. Proponents of leaving the blisters untouched often cite that it prevents wound desiccation and bacterial colonization.^4^ To counter that point, leaving blisters intact that are large and cumbersome prevent motion, which can lead to future stiffness and disability. Ibrahim et al^6^ recently have published a protocol for severe frostbite that includes the use of tissue plasminogen activator (TPA), which may demonstrate benefit and decrease amputation rates. However, the use of TPA has not been widely accepted for this injury. Future randomized clinical trials may help establish indications for TPA use in selected patients.

After 6 weeks of daily wound care and intensive occupational hand therapy, our patient was again living entirely independently, able to perform all self-care and activities of daily living.

**Figure:**